# Use of induced pluripotent stem cell derived neurons engineered to express BDNF for modulation of stressor related pathology

**DOI:** 10.3389/fncel.2014.00316

**Published:** 2014-10-14

**Authors:** Gele Liu, Nazneen Rustom, Darcy Litteljohn, Jessica Bobyn, Chris Rudyk, Hymie Anisman, Shawn Hayley

**Affiliations:** Hayley Lab, Department of Neuroscience, Carleton UniversityOttawa, ON, Canada

**Keywords:** stem cell, depression, BDNF, neurogenesis, stress

## Abstract

Combined cell and gene-based therapeutic strategies offer potential in the treatment of neurodegenerative and psychiatric conditions that have been associated with structural brain disturbances. In the present investigation, we used a novel virus-free re-programming method to generate induced pluripotent stem cells (iPSCs), and then subsequently transformed these cells into neural cells which over-expressed brain derived neurotrophic factor (BDNF). Importantly, the infusion of iPSC derived neural cells (as a cell replacement and gene delivery tool) and BDNF (as a protective factor) influenced neuronal outcomes. Specifically, intracerebroventricular transplantation of iPSC-derived neural progenitors that over-expressed BDNF reversed the impact of immune (lipopolysaccharide) and chronic stressor challenges upon subventricular zone adult neurogenesis, and the iPSC-derived neural progenitor cells alone blunted the stressor-induced corticosterone response. Moreover, our findings indicate that mature dopamine producing neurons can be generated using iPSC procedures and appear to be viable when infused *in vivo*. Taken together, these data could have important implications for using gene-plus-cell replacement methods to modulate stressor related pathology.

## INTRODUCTION

Just as is the case with neurodegenerative diseases, psychiatric conditions such as major depression, schizophrenia and post-traumatic stress disorder have been associated with structural brain changes, including reductions of adult neurogenesis, dendritic arborization and hippocampal volume (Andreasen et al., 2011; Kroes et al., 2011; Lee et al., 2013; Na et al., 2014). Moreover, it is well established that psychological and immunological stressors can promote structural brain changes, including altered neurogenesis and dendritic branching, along with marked hormonal variations (e.g., glucocorticoids; Anisman et al., 2008; Hayley, 2011). Accumulating evidence suggests that the deficiencies in growth factors, most notably brain derived neurotrophic factor (BDNF), likely contribute to the aberrant structural changes (Hasbi et al., 2009; Hayley and Litteljohn, 2013; Vithlani et al., 2013). Indeed, this growth factor is a critical mediator of neuroplasticity and neuroprotection, and has been implicated in many neurodegenerative and psychiatric disorders (Nagahara and Tuszynski, 2011; Autry and Monteggia, 2012). In fact, virtually all effective anti-depressant treatments influence BDNF and enhance neuroplasticity (Saarelainen et al., 2003; Schmidt and Duman, 2007, 2010). Given that BDNF does not cross the blood–brain barrier and can have deleterious systemic effects (Makar et al., 2009; Pilakka-Kanthikeel et al., 2013), finding novel means of delivering BDNF to the brain over long time periods is of great importance.

One exciting currently evolving approach to the potential delivery of trophic factors and the engagement of “repair” mechanisms within the brain involves the use of stem cell based technologies. In particular, the transcription factors, Oct4 (octamer-binding transcription factor 4), Sox2 [SRY (sex determining region Y)-box 2], Klf4 (Krüppel-like factor 4), and c-Myc (cellular myelocytomatosis oncogene), are capable of re-programming adult fibroblasts into induced pluripotent stem cells (iPSCs), which can be further differentiated into targeted functional types of cells, including neurons (Breton et al., 2013; Faiz and Nagy, 2013). Importantly, iPSC derived cells have been posited to have clinical potential as a cell replacement strategy or means of inducing neuroplasticity in neurodegenerative disorders, such as Parkinson’s disease, Alzheimer’s disease and spinal cord injury, and possibly even neuropsychiatric illnesses (Vaccarino et al., 2011; Yagi et al., 2012; Faiz and Nagy, 2013).

Genetic- (e.g., gene targeting) and- cellular restoration-based therapies (e.g., iPSCs) have been considered as totally independent, and even mutually exclusive, approaches to treatment, even though they have obvious commonalities (Müller et al., 2006). With regard to the brain, both approaches seek to modify neural circuitry or protein levels either by (1) altering existing gene expression within brain cells, or (2) promoting the integration of novel cells into existing/damaged neural circuits. In the present investigation we combined these two strategies by over-expressing BDNF in iPSC-derived immature neural cells and sought to determine whether central infusion of these cells would modify the impact of stressor exposure. We chose a compound stressor comprising lipopolysaccharide (LPS) plus a chronic psychological stressor regimen, which previously elicited robust hypothalamic–pituitary–adrenal (HPA), behavioral and neurochemical changes (Gibb et al., 2011). Our current methods also utilized a non-viral method for the reprogramming of fibroblasts into iPSCs, thereby minimizing the potential for tumor formation (as was previously reported with retroviral methods; Wernig et al., 2008). Finally, we also tested the ability of our procedures to create mature dopamine producing neurons and whether these cells would survive, at least for the short term, when infused *in vivo*. Our “enhanced gene-cell approach” permits the exploration of potential additive or synergistic effects of iPSC-derived cells together with gene-directed expression of BDNF.

## MATERIALS AND METHODS

**FIGURE 1:**
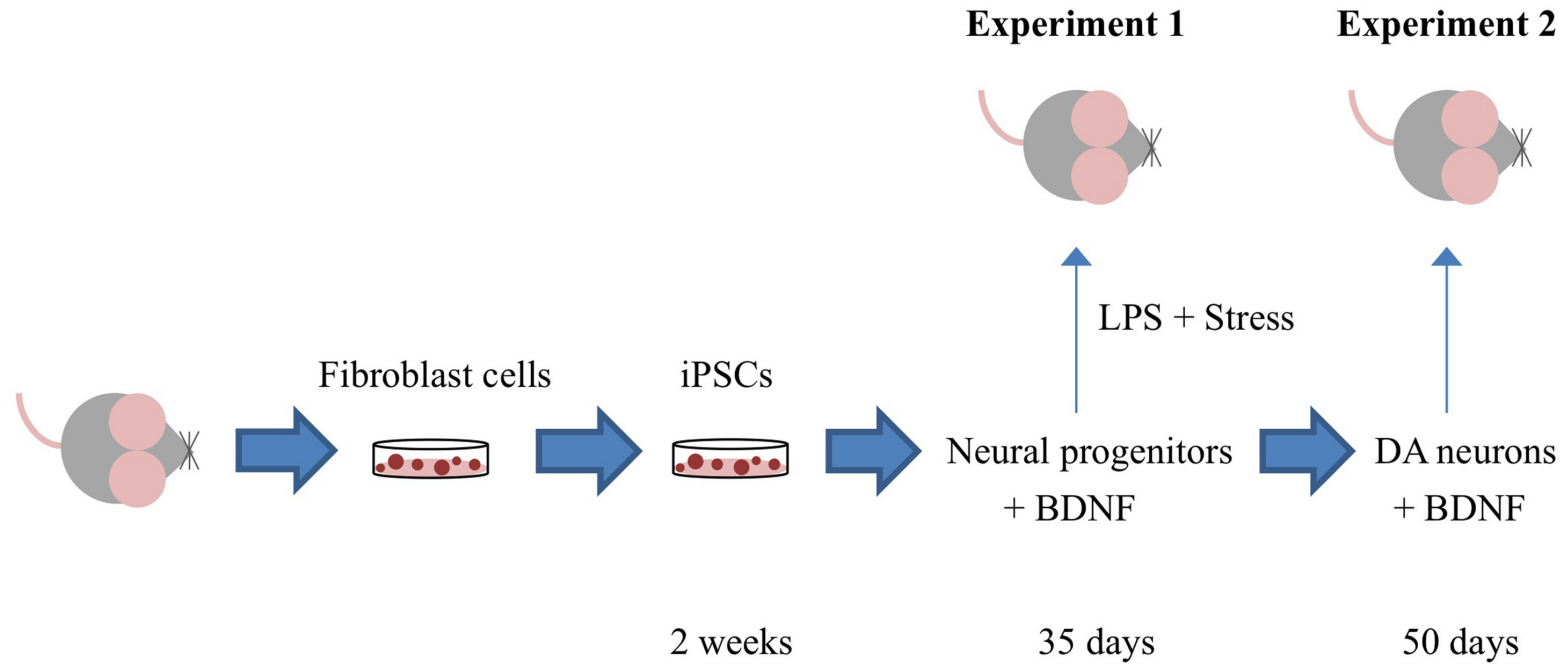
**Outline of the specific experiments conducted.** Adult mouse fibroblast cells were reprogrammed into induced pluripotent cells (iPSCs) using the vector 20866 containing the c-Myc, Klf4, Oct4, and Sox2 genes. iPSCs were further sequentially transformed into immature neural progenitor cells (after 35 days in culture) and mature dopamine neuronal cells (after 50 days in culture). In Experiment 1, iPSC-derived neural progenitor cells alone or with the additional cDNA BDNF were infused in order to determine their ability to reverse the impact of psychological and immunological [lipopolysaccharide (LPS)] stressor exposure. Experiment 2 involved the generation of iPSC-derived mature DA neurons (with and without cDNA BDNF transfection) and assessment of their survival following central infusion.

**Figure 1** depicts the timing of the experiments, which are described in detail in the ensuing sections.

### VECTOR CONSTRUCTION

We utilized a non-viral vector, termed pCAG2LMKOSimO (vector 20866, Addgene), for reprogramming somatic fibroblasts into iPSCs (Kaji et al., 2009). This vector takes advantage of producing virus-free, factor-removable iPSCs by applying a single plasmid with a 2A-peptide-linked reprogramming cassette, c-Myc-Klf4-Oct4-Sox2-(MKOS)-IRES-mOrange, flanked by loxP sites. These reprogramming genes are transcribing from the universally expressed synthetic CAG enhancer/promoter (Niwa et al., 1991; Szymczak et al., 2004). Contained in this vector are the coding sequences of four transcription factor genes, namely c-Myc, Klf4, Oct4, and Sox2, which together comprise the reprogramming cassette; vector 20866 also contains the mOrange marker for gene expression.

**FIGURE 2:**
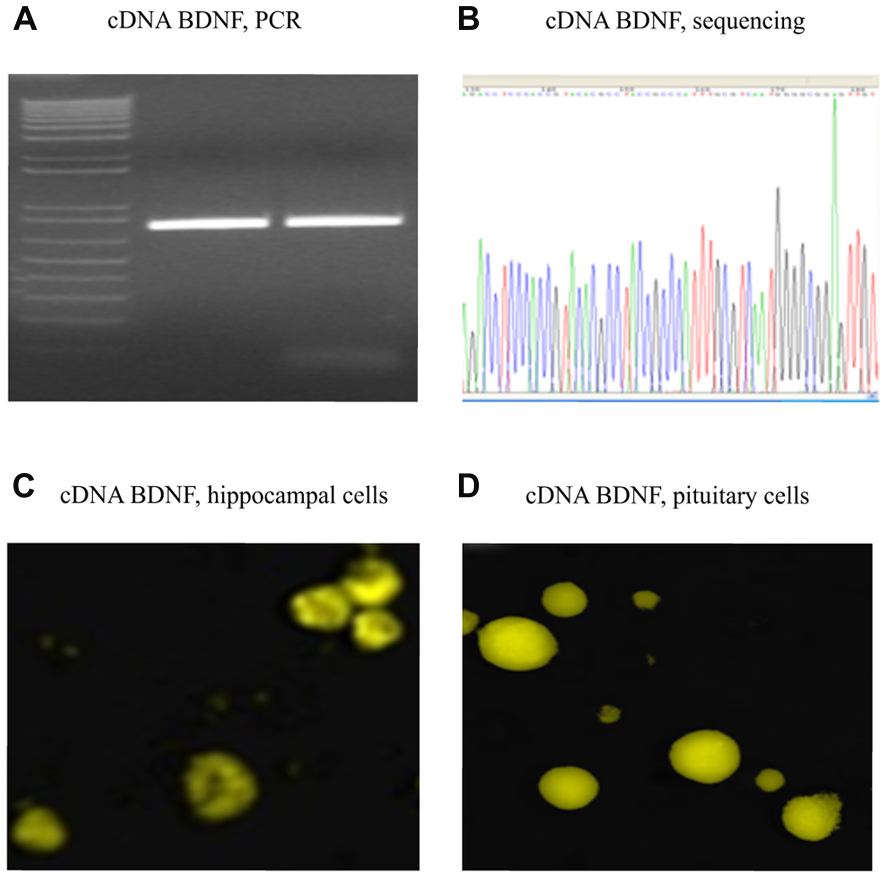
**Verified full-length gene sequence of BDNF cDNA.** A 750 bp PCR product for cDNA BDNF was present on a 1% agarose gel **(A)**. The full-length cDNA BDNF was then cloned into a pZsYellow1-N1 Vector and verified by sequencing **(B)**, as well by immunofluorescent detection (anti-BDNF antibody from Abcam ab620; 1:150) in hippocampal **(C)** and pituitary gland **(D)** primary cell cultures of adult CD1 mice.

The full sequence of 750 bp cDNA for BDNF from mus musculus (GenBank BC034862) was constructed using polymerase chain reaction (PCR; **Table 1** for the Primer Lists) and cloned into the pZsYellow1-N1 Vector (Clontech 632445) at the ligation sites of XhoI and SalI. cDNA BDNF expression was driven by the human cytomegalovirus (CMV) enhancer/promoter. The complete mouse cDNA for BDNF was verified by Sanger Sequencing, using Applied Biosystem’s 3730xl DNA Analyzer technology at The McGill University Innovation Centre (**Table 1** for the Primers). Full cDNA BDNF was transfected into adult mouse primary hippocampal or pituitary cells, as well as adult rat primary hippocampal cells for verification of gene expression (**Figure 2**). Primary hippocampal and pituitary cell cultures were prepared from adult (aged 2 months; CD1) mice.

**Table 1:** Brain derived neurotrophic factor primers for cloning PCR product or sequencing complete cDNA construct.

Genes		Primers
Full cDNA BDNF	Forward	accaggtgaCTCGAGgaagagtgatgaccatcc
	Reverse	cataaatccactaGTCGACtcttccccttttaatgg
Sequencing BDNF	BDNF-r1	CAAGTTGCCTTGTCCGTG
	BDNF-r2	CCTGGGCCCATTCACGCTCTCCAGAG
	BDNF-f1	CATCCTTTTCCTTACTATGGTTATTTC
	BDNF-f2	CAGTCAAGTGCCTTTGGAG
	BDNF-f3	CAAGTGTAATCCCATGGGTTAC

### TISSUE PREPARATION AND CELL CULTURE

Fibroblasts were isolated from tail-tips of adult (aged 2 months; CD1) mice and collected in Dulbecco’s Modified Eagle Medium: Nutrient Mixture F-12 (DMEM/F12, Invitrogen 10565-018). Culture vessels were coated with poly-D-lysine (final coating concentrations from 1 μg/cm^2^ of surface area, Millipore A-003-E) and laminin (final coating concentrations from 1 μg/cm^2^ of surface area, Invitrogen 23017-015).

Tissues were placed on a plate with 4 mL of the Hibernate®;-E Medium without Ca^2+^ (BrainBits LLC, HE-Ca) or DMEM/F12. Pieces of tissue were then enzymatically digested using 2 mg/mL of filter-sterilized papain (Worthington, Cat. no. LS003119) at 37°C for 45 min. Subsequently, these were removed and spun down and the supernatant re-suspended in 5 mL of complete Hibernate®;-E medium (BrainBits LLC) or knockout DMEM (Invitrogen 10829-018). The tubes were further centrifuged for 4 min at 200 × *g*, after which supernatants were removed and cell pellets re-suspended in 41.5 mL DMEM/F-12 (Invitrogen 10565-018). A total of 50 mL of the medium was prepared by adding a further 7.5 mL knockoutTM serum replacement (Invitrogen 10828-028), 0.5 mL L-glutamine (Invitrogen 25030-081), 0.5 mL MEM Non-Essential Amino Acids Solution (NEAA; Invitrogen 11140-050), 50 μl of 10 μg/mL (final 10 ng/mL) bFGF (Fibroblast growth factors, Invtrogen 13256-029) and 91 μl (final 0.1 mM) β-mercaptoethanol (Invitrogen 21985-023). Cells were incubated at 37°C in a humidified atmosphere of 5% CO_2_ in ambient air, and were freshly fed every third day with the appropriate medium.

### REPROGRAMMING GENES AND INDUCTION OF PLURIPOTENCY

Once the aforementioned cells were cultured to 90% confluence, we transfected the vector 20866 and/or cDNA BDNF using lipofectamine®; 2000 Transfection Reagent (Invtrogen 11668-027), according to the manufacturer’s instructions. Cells with/without vector 20866 and/or BDNF cDNA were continually cultured with fresh media. The cluster appearance of stem-cell-like colonies expressing the mOrange-positive marker were distinguished at Days 5–6 following transfection. The iPSCs were confirmed by means of positive alkaline phosphatase live stain (Invitrogen A14353), as well as an expression of stem cell stage-specific antigens: SSEA-1, SSEA-3, Oct-4, and TRA-1-81 (Millipore SCR002). The iPSCs with the mOrange-positive marker were further recognized during Days 17–20 by live staining with TRA-1-81, and then these were transported to cultures containing a Neural Induction Medium in newly coated-plates or wells comprising a 0.1% gelatin attachment factor solution (Invitrogen S-006-100).

The cDNA transfection day was denoted as Day 0, and from Days 1 to 14 the medium was changed and replaced with complete N2B27 Medium (Invitrogen 11330-057, plus N-2 Supplement 17502-048, B-27®; Supplement 17504-044, knockoutTM serum replacement, L-glutamine, NEAA, β-mercaptoethanol), supplemented with a CHALP cocktail (containing MEK, TGF-b and GSK3β inhibitors, along with human leukemia inhibitory factor) which significantly improved reprogramming efficiency (Yu et al., 2011). During Days 15–20, the medium was changed to Essential 8TM Medium (Prototype), and was replaced every other day.

### NEURAL PROGENITOR CELL INDUCTION

During Days 21–28, the undifferentiated iPSCs formed neural progenitor cells following culture in the Neural Induction Medium. This medium consisted of 50 mL Neurobasal®; medium with bFGF, heparin solution (Sigma H3149, 50 μl of 2-mg/mL), N-2 supplement, glutaMAX^TM^-I supplement, NEAA, knockout^TM^ serum replacement, and β-mercaptoethanol. The medium was changed every other day.

### MATURE DOPAMINE NEURONAL DIFFERENTIATION

To direct the neural progenitor cells to differentiate into dopamine neural progenitors, cultures were fed every other day (Days 29–35) with Dopamine Neuronal Progenitor Medium, containing 100 ng/mL FGF-8b (Invtrogen PHG0271) and 200 ng/mL sonic hedgehog (SHH, R&D systems 1314-SH-025), as well as Neurobasal®; medium, heparin solution, N-2 supplement without vitamin A (Invtrogen 12587-010), and NEAA.

Subsequently, from Days 36 to 50 the dopamine neural progenitor cells were further differentiated into mature dopamine neurons by culturing in the Dopamine Neuronal Differentiation Medium every other day. This medium comprises: recombinant human BDNF (Invtrogen PHC7074, 50 μl of 25-μg/mL); recombinant human GDNF (Invtrogen PHC7045, 50 μl of 20-μg/mL); ascorbic acid (Sigma A4403, 50 μl of 200 mM); dcAMP (Dibutyryl cyclic-AMP; Sigma D0627, 50 μl of 1-mM) in 50 ml Neurobasal®; medium containing heparin; N-2 supplement without vitamin A (Invitrogen 12587-010); and NEAA.

### STEREOTAXIC INFUSION OF iPSC-DERIVED CELLS

12-month old male CD1 mice were used as experimental animals for intracerebroventricular infusion. All procedures were approved by the Carleton University Committee for Animal Care and were conducted in adherence to guidelines set out by the Canadian Council for the Use and Care of Animals in Research. Briefly, mice were anesthetized with oxygen-enriched isoflurane, and placed in a stereotaxic apparatus. All cannulae were implanted according to the following stereotaxic coordinates: 0.14 mm anterior to bregma, 0.75 mm lateral from the mid-sagittal suture, and 2.35 mm ventral from a flat skull surface. Animals were given 1 week to convalesce before experimentation. Intracerebroventricular infusion volume was 4 μL (5 × 10^5^) of the reprogrammed neural cells. Infusions were delivered through polyethylene tubing connected to a microliter syringe (Hamilton; Reno, NV, USA) and syringe pump (Harvard Apparatus Pico Plus; Holliston, MA, USA). All infusions were delivered at a rate of 0.4 μL/min, for a total of 10 min, followed by a 5-min pause to ensure proper delivery. Infusions were conducted between 08.30 and 14.00 h to avoid variability attributed to diurnal variations. Furthermore, animals were unrestrained during the infusion process to minimize stress.

### CENTRAL INFUSION AND STRESSOR PROCEDURES

#### Experiment 1

For this study, mice were infused with LPS (2 μg dissolved in 4 μL saline) or saline. At 24 h following the intracerebroventricular infusion, a 3-week stressor regimen began, wherein mice received a different stressor twice daily (as described in the upcoming section). At the end of the first week of stressor exposure regimen and following the LPS/saline infusion, mice received a second single intracerebroventricular infusion of either: (1) HBSS (vehicle), (2) reprogrammed neural progenitors (iPSCs cultured for 35 days in differentiation medium) alone, or (3) reprogrammed neural progenitors plus complete cDNA BDNF. As well, a separate group of HBSS infused animals did not receive any LPS or stressor treatment. All animals were sacrificed 2 weeks following the second infusion (i.e., at the end of the 3 weeks of stress).

The stressor exposure occurred twice daily (morning and afternoon) and lasted for 3 weeks. The chronic stressor regimen included the following stressors: restraint in semicircular Plexiglas tubes (4 × 12 cm) with tails taped to prevent mice from turning (15 min); restraint in tight-fitting triangular bags with a nose-hole for breathing (15 min); exposure to social stressors (interactions but not fighting) by placing mice in a large cage (40 cm × 25 cm × 15 cm) divided into separate quadrants with three non-experimental mice (60 min); tail hanging (30 s); placement in an empty cage without sawdust or nesting (60 min); lights on during dark phase; intermittent background noise in an isolated chamber (30 cm × 14 cm × 15 cm, 40 dB, 10 min); and 30° cage tilt (60 min). Following the first (morning) daily stressor, mice were returned to their home-cage until application of the second (afternoon) stressor. Due to the nature of the stressor paradigm, animals that received the chronic stressor regimen were housed in holding rooms separate from, but otherwise identical to, their non-stressed counterparts.

#### Experiment 2

Compared to immature neural progenitor cells, mature neurons have a greatly reduced capacity for plasticity. There is therefore a concern that the transformed mature dopaminergic neurons might not survive after being transplanted into the complex microenvironment of the brain. To examine this possibility, iPSC-derived mature dopaminergic neurons, either alone or additionally transfected with BDNF, were transplanted into adult mouse lateral ventricles and infusion of the medium, HBSS, served as the experimental control. Twenty-four hours after cell transplantation (or vehicle infusion), cortical and hippocampal brain regions were collected. These regions were of interest given their close proximity to the infusion site, along with their importance in mediating responses to stressors. As it was previously reported that neural stem cells injected into the striatum of tumor bearing rats migrated at a rate of ∼175 μm/min (Kim et al., 2010), 24-h should be sufficient time for some degree of migration of the iPSC-derived neurons.

For this study, (see **Figure 1**), mice did not receive any LPS or stressor treatments. However, animals did get a single intracerebroventricular infusion of either HBSS (control) or iPSC-derived mature dopaminergic neurons (cultured for 50 days in differentiation medium), either alone or additionally transfected with BDNF. Twenty-four hours after cell transplantation (or vehicle infusion), cortical and hippocampal brain regions were collected in order to ascertain whether the mature neurons survived *in vivo*.

### QUANTITATIVE REAL-TIME PCR (qRT-PCR)

Total RNA was isolated using a GeneJET RNA Purification kit (Thermo scientific K0732) and then quantitative real-time PCR (qRT-PCR) was carried out using a LightCycler®; 480 RNA Master Hydrolysis Probes kit (Roche 04991885001) according to the manufacturer’s instructions. The TaqMan-gene-specific primers were designed using Integrated DNA Technologies’ PrimeTime qPCR Assay web application. TaqMan-gene-specific primers were designed using an Integrated DNA Technologies proprietary software program. All experiments were normalized for PCR sample loading and reaction, and data represent one of three independent experiments for ct (threshold cycle) plots. All gene expression values were normalized to total levels of the control housekeeping gene, GAPDH, whose primers are presented in **Table 1**.

### HIGH PERFORMANCE LIQUID CHROMATOGRAPHY (HPLC)

Cell samples were homogenized by ultrasonic disruption in a 500 μl solution containing 0.1 mM NaEDTA, 0.3 M monochloroacetic acid, 1% methanol in high performance liquid chromatography (HPLC) grade water, with DHBA as an internal standard. The level of protein was determined with Pierce BCA Protein Assay Kit (Thermo Scientific 23225). Homogenated samples were centrifugated at 10000 rpm at 4^o^C and 50 μl of supernatant was injected, at a flow rate of 1 ml/min, into the HPLC system (Agilent 1100) with electrochemical detector (DECADE SDC) and Eclipse XDB-C-8, 4.6-150 mm column. The mobile phase consisted of 90 mM sodium dehydrogenate Phosphate, 1.1 mM 1-octane sulfuric acid, 50 mM EDTA (1 ml/2l of 100 mM solution), and acetonitrile 10%, citric acid 50 mM, potassium chloride 5 mM, and HPLC-grade water.

### IMMUNOHISTOCHEMISTRY

For the assessment of *in vitro* stem cell markers, alkaline phosphatase live stain (Invtrogen, A14353) and Embryonic Stem (ES) Marker Sample Kits (Millipore, SCR002) were applied. The latter kit contains monoclonal antibodies for the detection of the cell-surface stage-specific embryonic antigens (SSEA-1, SSEA-3, and SSEA-4), as well as expression of TRA-1-60, TRA-1-81 antigens and Oct-4. Neuronal cells were detected using immature neuronal cell marker anti-DCX antibody (Invitrogen 48-1200), the mature neuronal cell marker anti-MAP2 antibody (Abcam ab32454), the dopamine neuronal cell marker tyrosine hydroxylase (TH) antibody (ImmunoStar 22941). Briefly, the primary antibodies were diluted at 1:25 ratio in blocking solution (1× PBS/4% normal goat serum/0.3% Triton^TM^ X-100), and then incubated for 1 h at room temperature following three washes with 1× Rinse Buffer. Further, samples were incubated with fluorescein isothiocyanate (FITC)-labeled secondary antibodies for 1 h at room temperature. Fluorescence images were visualized with an Olympus 1×2-UCB series microscope and MediaCybernetics imaging software. Living cell culture samples were assessed using a Zeiss Axiovert 40 CFL microscope and Lumenera Infinity software.

For assessment of adult neurogenesis, mice were sacrificed by perfusion with saline followed by 4% paraformaldehyde (PFA). Brains were post-fixed in a solution of 30% sucrose in 4% PFA and were then flash-frozen and cryostat sliced into 20-μm coronal sections containing the hippocampus and subventricular zone (SVZ). The hippocampal levels collected were operationally defined as early/rostral (bregma -1.22 to -1.82), middle (bregma -1.82 to -2.46) and late/caudal (bregma -2.46 to -2.92), as we have previously reported (Seguin et al., 2009). The SVZ levels were collected between bregma levels 0.26 to -0.10. All tissue was immersed in heated tri-sodium citrate buffer (pH 8.5) for antigen retrieval following preliminary washes with phosphate buffered saline (PBS). Subsequently, tissue was blocked for 1 h with 0.1 M tris-buffered saline containing 0.1 M PBS containing 0.1% sodium azide, 0.3% triton-X, and 2% bovine serum albumin (BSA). Sections were incubated with primary antibody for doublecortin (DCX; Life Technologies 48-1200, 1:500) overnight at 4^o^C. Thereafter, sections were further incubated for 2 h at room temperature with biotin anti-rabbit (Jackson ImmunoResearch Laboratories; 1:1000). Primary and secondary antibodies were diluted in 0.01 mol/l PBS (pH 7.3) containing 2% BSA with 0.3% Triton X-100 and 0.01 sodium azide. Sections were further incubated in horseradish peroxidase-conjugated streptavidin tertiary antibody (1:1000; Jackson ImmunoResearch) at room temperature for 2 h. Antibodies were then visualized by incubation with DAB (Sigma-Aldrich) for 10 min on a shaker table.

All quantitative analyses were performed in a blinded fashion with the total number of bilaterally labeled DCX-positive cells counted within the rostral, middle and caudal portions of the dentate gyrus or between the SVZ bregma levels 0.26 to -0.10, as mentioned above. Using a 20× magnification, the number of immuno-positive cells per section was manually counted for each of the three hippocampal levels, as well as the overall total number of DCX+ cells within the sampled SVZ region. Five different slides (with four sections/slide) for each animal were quantified and the average number of DCX+ neurons determined for each animal and treatment comparisons made.

### WESTERN BLOT

Briefly, cells or tissues were lysed and sonicated for 2 s and protein concentrations were measured by a standard BCA assay (Pierce). All samples were heated in Laemmli buffer and 15 μg of total protein subsequently loaded on a 12.5% SDS-PAGE gel. Blots were then probed with a rabbit polyclonal antibody (Invitrogen 48-1200) against the immature neuronal cell marker, DCX. Secondary antibodies that were HRP conjugated were then applied and proteins visualized by ECL treatment and subsequent exposure to Kodak film. For loading controls, all lanes were loaded with beta actin antibody (abcam, ab8227) at 1/5000 dilution.

### CORTICOSTERONE ANALYSES

Trunk blood was collected in tubes containing 10 μg EDTA, centrifuged at 3600 rpm for 8 min, and 50 μL of plasma was then collected for determination of corticosterone levels. Plasma was immediately frozen at –80^o^C until analyses. Corticosterone levels were measured using a commercial radioimmunoassay kit (ICN Biomedicals, CA, USA, Cat .no 07120002). Inter-assay variability was avoided by assaying all samples (in duplicate) within a single run.

### STATISTICAL ANALYSES

A StatView (SAS Institute, version 6.0) statistical software package was used for all computations. Behavioral, corticoid and immunohistochemical measures were analyzed for statistical significance using an appropriate analysis of variance (ANOVA), wherein treatment infusion and stressor/LPS exposure vs. no stress were treated as independent variables. Any significant findings were followed up using Bonferonni analyses.

## RESULTS

### OUTLINE OF EXPERIMENTS

The outline of the specific experiments conducted is presented in **Figure 1**. Briefly, these comprised studies in which (1) iPSCs derived from re-programmed mouse tail-tip fibroblasts were differentiated into neural progenitor cells (that expressed DCX) and infused into adult mouse lateral cerebral ventricles. After a 3-week stressor challenge that comprised a combined single LPS infusion followed by chronic psychogenic/neurogenic stressor exposure, we assessed whether the transplanted cells provided protection against stressor-induced changes in adult neurogenesis and plasma corticosterone (Experiment 1) and (2) iPSC-derived neural progenitors were further differentiated *in vitro* into mature neurons (that expressed TH and MAP2 and produced dopamine), and the *in vivo* survival of these cells was assessed 24-h after intracerebroventricular transplantation (Experiment 2).

### VERIFICATION OF BDNF cDNA

Polymerase chain reaction product for full-length BDNF cDNA was cloned into a pZsYellow1-N1 Vector, and verified by sequencing (**Figures 2A,B**). Its gene expression *in vitro* was determined by immunofluorescent detection using anti-BDNF antibody in hippocampal (**Figure 2C**) and pituitary gland (**Figure 2D**) primary cell cultures from adult CD1 mice.

### VIRUS-FREE *IN VITRO* REPROGRAMMING OF MATURE FIBROBLAST CELLS INTO iPSCs

**FIGURE 3:**
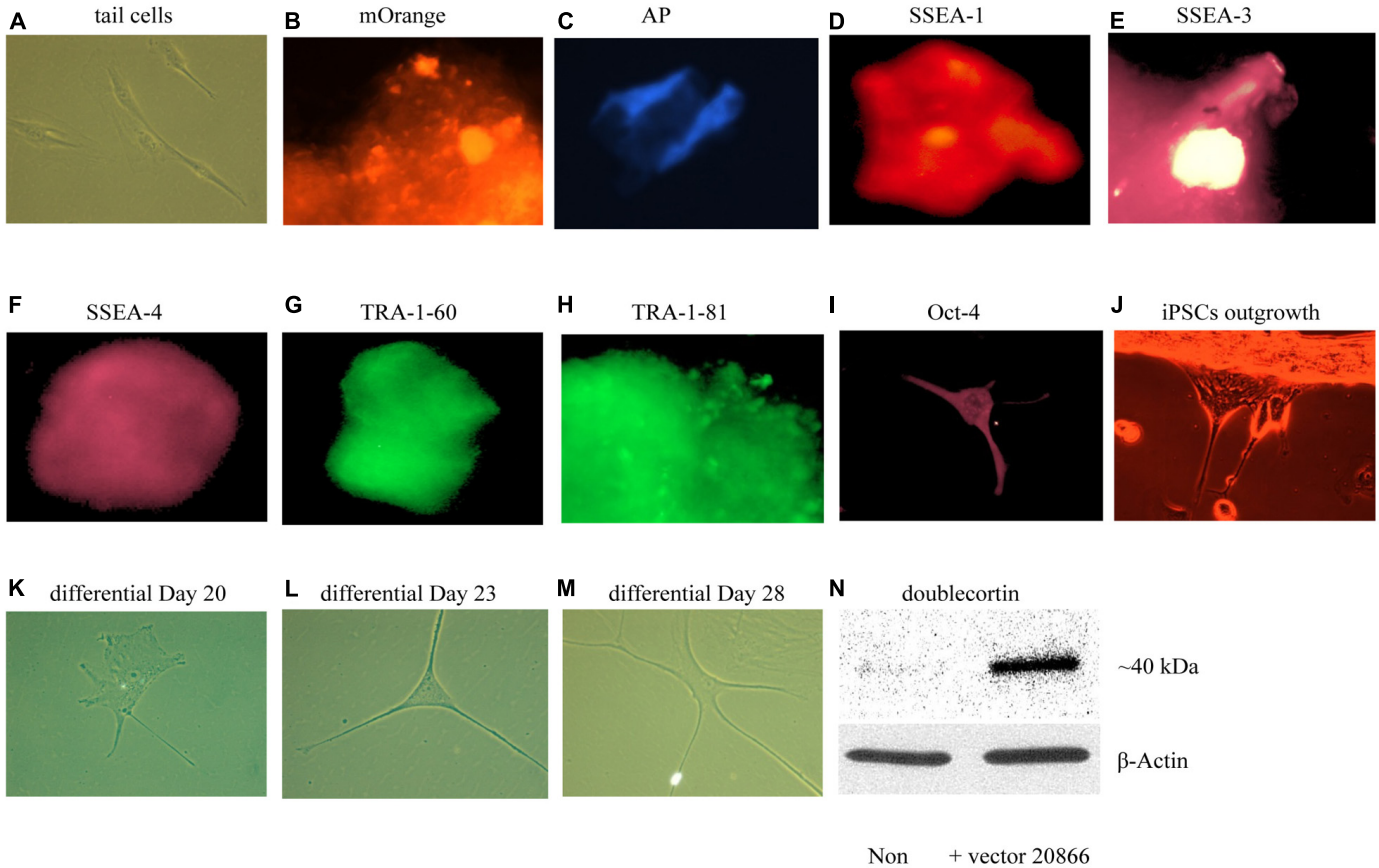
***In vitro* iPSCs and neural progenitor cells produced from adult mouse fibroblasts (see Materials and Methods).** The panels show that control (absence of the 20866 vector) tail cells still retain their original shape on Day 47 after culturing **(A)**. The presence of the mOrange marker suggests that the fibroblasts have successfully integrated the 20866 plasmid **(B)**. The iPSCs were further characterized by an alkaline phosphatase live stain **(C)**, as well as staining of the three cell-surface stage-specific embryonic antigens: SSEA-1 to -4 **(D–F)**, TRA-1-60 **(G)**, TRA-1-81 **(H)**, and Oct-4 **(I)**. By Day 18, iPSCs began displaying projections **(J)** and further differentiation was clearly evident by Days 20, 23, and 28 **(K–M)**, respectively. By the later time point **(M)**, the cells had morphology consistent with that of neural progenitor cells and displayed a robust induction of the immature neuronal marker, doublecortin **(N)**.

We first sought to optimize existing *in vitro* reprogramming methods established by Kaji et al. (2009). As mentioned in the methods, adult mouse tail-tip fibroblast cell cultures (from 2-month old mice) were transfected with the non-viral vector 20866 containing the four reprogramming transcription factors: Oct4, Sox2, Klf4, and c-Myc. The iPSC identity was established by the expression of the fluorescent vector marker, mOrange (**Figure 3B**), and further verified using a numbers of markers – alkaline phosphatase live stain (**Figure 3C**), as well as the cell-surface, SSEA-1 (**Figure 3D**), SSEA-3 (**Figure 3E**), SSEA-4 (**Figure 3F**), TRA-1-60 (**Figure 3G**, tumor rejection antigen 1-60), TRA-1-81 (**Figure 3H**), and Oct-4 (**Figure 3I**). Thus, it appeared that successful reprogramming occurred. Furthermore, the colonies were picked between Days 17–20, and seeded on gelatin-coated plates (Attachment Factor is sterile 1× solution containing 0.1% gelatin, Invitrogen S-006-100).

### EXPERIMENT 1: GENERATION OF NEURAL PROGENITOR CELLS FROM iPSCs

Upon successfully demonstrating the ability of our non-viral reprogramming system to generate pluripotency *in vitro*, our next step was to produce cells of a neuronal phenotype. In particular, we were initially most interested in generating neural progenitor cells, given their relatively high degree of plasticity. Using a specific neural induction medium, iPSCs were differentiated into neural progenitor cells between Days 20 and 35. The neural morphology of these cells was quite apparent, with clear soma and distinct neural processes being progressively evident over time (**Figures 3A–M**). Moreover, the immature neuronal marker, DCX, was clearly up-regulated in these cells, indicating that these newly generated neuronal precursors are no more than 3 weeks of age and have not yet reached a mature stage (**Figure 3A–N**).

### iPSC-DERIVED NEURAL PROGENITOR CELLS BLUNTED THE *IN VIVO* EFFECTS OF LPS + STRESS

**FIGURE 4:**
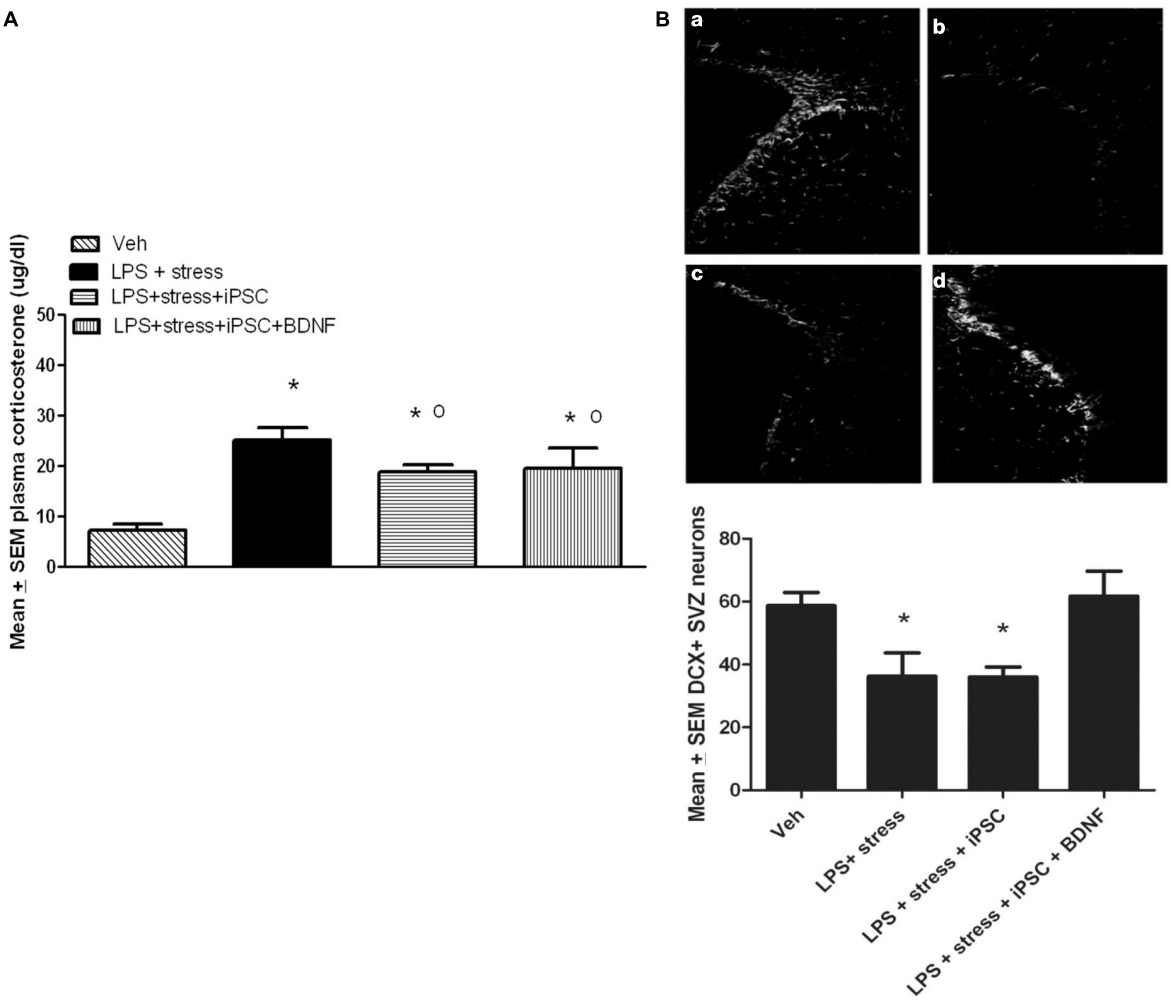
**As shown in the bar graph on the left **(A)**, the single icv LPS infusion (2 μg) plus a 3-week twice-daily different stressor challenge (LPS + stress) increased plasma corticosterone, relative to vehicle treatment (Veh).** Icv infusion of either the iPSC-derived neural progenitor cells alone (LPS + stress + iPSC) or in combination with the cDNA BDNF (LPS + stress + iPSC + BDNF) attenuated the plasma corticosterone rise induced by the LPS + stress treatment. The photomicrograph on the right **(B)** displays doublecortin (DCX) immunoreactivity in the subventricular zone (SVZ) in response to vehicle (a), LPS + stressor exposure (b), LPS + stressor exposure + iPSC-derived neural progenitor cells (c), or LPS + stressor exposure + iPSC-derived neural progenitors + BDNF cDNA (d). As quantified in the bottom bar graph, the LPS + stressor exposure alone or in combination with the iPSC-derived neural progenitor cells significantly reduced the number of DCX+ cells within the SVZ (*p* < 0.05). However, infusion of the iPSC derived neural progenitor cells that were transfected with cDNA BDNF completely prevented this DCX reduction. *p < 0.05 relative to Veh treatment, o *p* < 0.05 relative to LPS + stress treatment.

A single factor ANOVA indicated that the treatments were associated with significant plasma corticosterone changes, *F*(3,16) = 9.00; *p* < 0.001 (**Figure 4A**). Follow-up Bonferonni corrected tests confirmed that the LPS-stressor challenge provoked a robust and significant rise of corticosterone (*p* < 0.05) relative to non-stressed mice. As shown in **Figure 4A**, mice that were transplanted with the neural progenitors, regardless of whether the cells had been further transfected with BDNF, displayed a significant attenuation of LPS + stressor-induced corticosterone levels (*p* < 0.05), relative to animals that only received the LPS + stressor treatment. Yet, all three LPS + stressor treatment group (regardless of iPSC infusion) still had corticosterone levels that exceeded that of the non-stressed controls (*p* < 0.05).

No significant differences were observed between the treatment groups for the number of DCX positive neurons within the dentate gyrus region of the hippocampus. Indeed, the iPSC derived cellular infusions did not significantly affect the number of DCX+ neurons at either of the three different hippocampal levels sampled (data not shown). However, significant between-groups differences were evident within the SVZ, *F*(3,18) = 5.02, *p* < 0.05. As shown in **Figure 4B** and confirmed by follow up comparisons, LPS + stressor exposure provoked a significant reduction in the total number of DCX-expressing neurons within the sampled SVZ (*p* < 0.05), and this effect was totally prevented by co-treatment with the iPSC-derived neural progenitors that also expressed BDNF. Interestingly, infusion of iPSC-derived neural progenitors that did not co-express BDNF had no significant effect on DCX cells (**Figure 4B**), suggesting the importance of this trophic factor in modulating SVZ neurogenesis. It is likely that the SVZ and not the hippocampal neurons were affected given their proximity to the lateral ventricular site of infusion. Importantly, previous studies have indicated that SVZ neurons may act as a reservoir of cells that are especially sensitive to stressful events, often migrating toward sites of brain injury (e.g., following cerebrovascular stroke) and infection (Goings et al., 2008; Ahlenius et al., 2012).

### EXPERIMENT 2: GENERATION OF MATURE DOPAMINERGIC NEURONS IN CULTURE

**FIGURE 5:**
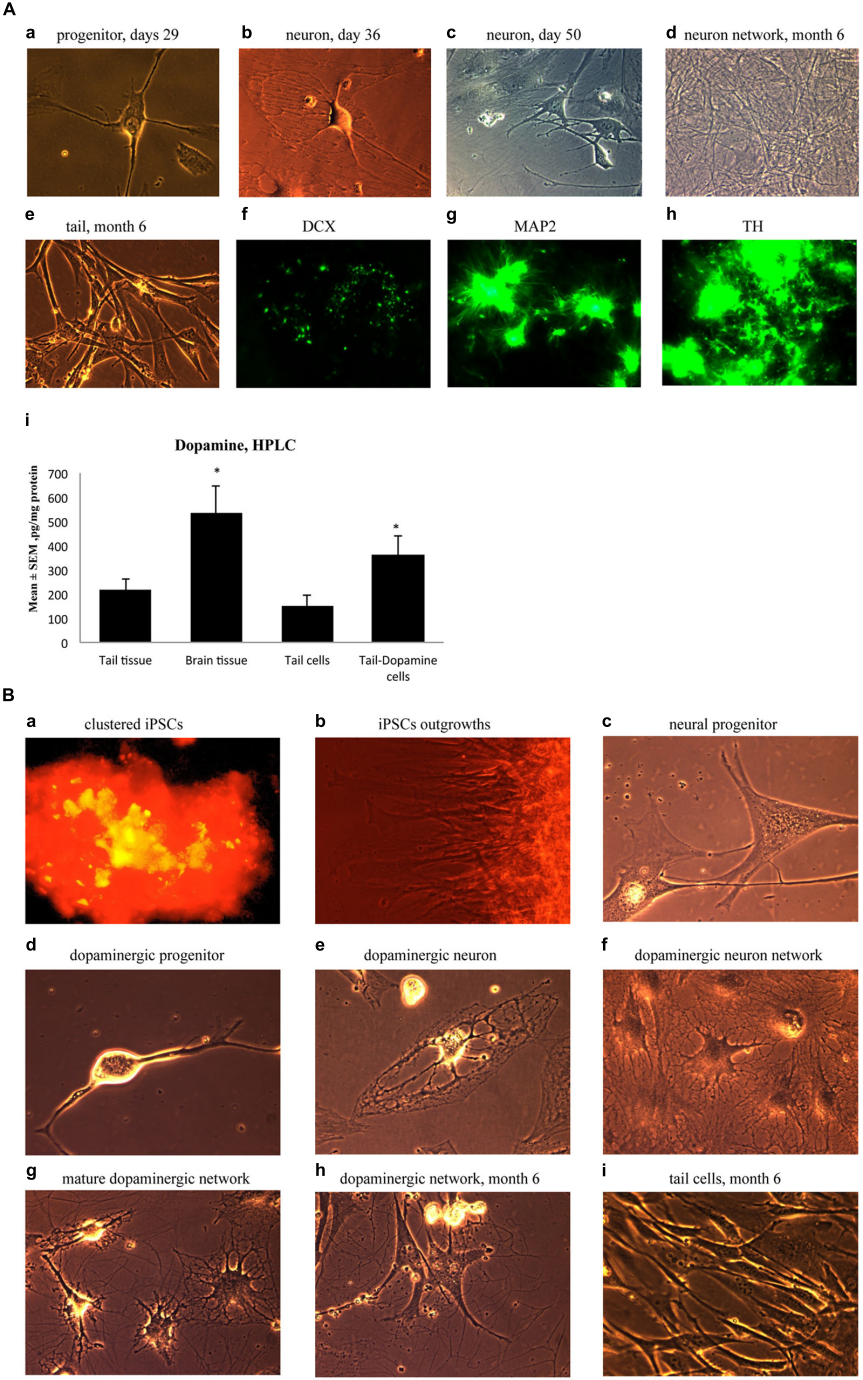
**(A)** Immature neuronal cells that were cultured in a Dopaminergic Neuronal Progenitor Medium began to show morphological signs of neuronal maturity between Day 29–50 (a,b,c) and after 6 months these cells displayed further signs of mature network formation (d). Importantly, control tail cells that were not subject to the reprogramming procedures kept their original shape at 6 months (e). As well, during this time, expression of the early immature neuronal cell marker, DCX, was greatly diminished (f), together with increased expression of the mature general neuronal cell marker, microtubule associated protein 2 (MAP2), (g), and specific dopaminergic neuronal marker, tyrosine hydroxylase (TH), (h) was evident. Further confirmation that the reprogrammed neurons were mature dopaminergic neurons was obtained using HPLC (i); wherein tail cells that were re-programmed (Tail-Dopamine cells) displayed significantly increased dopamine levels, compared to non-reprogrammed controls (Tail cells). Furthermore, dopamine levels in non-reprogrammed controls were, as expected, quite low (Tail tissue), whereas freshly dissected adult midbrain tissue had the expected highest concentration of dopamine (Brain tissue). *p < 0.05, relative to control tails. **(B)** Shows the time course for dopaminergic neuron derivation. The adult mouse (aged 2 months) fibroblasts were reprogrammed by vector 20866 with c-Myc, Klf4, Oct4, and Sox2 genes and an mOrange marker was used to detect gene expression after 2-weeks in cell culture (a). Induced pluripotent stem cells (iPSCs) began to show obvious outgrowths at Days 15 and 20 (b). The undifferentiated iPSCs were transformed into neural progenitor cells following culture in the Neural Induction Medium (see Materials and Methods) during Days 21–28 (c). The neural progenitor cells were further differentiated into dopaminergic neural progenitors with the Dopamine Neuronal Progenitor Medium from Days 29–35 (d). From Days 36 to 50, the cells were subsequently differentiated into mature dopaminergic neurons by culturing in the Dopamine Neuronal Differentiation Medium (e,f,g). The mature dopaminergic neurons were grown for up to 6 months (h), and were visibly easy to distinguish from the original un-reprogrammed adult fibroblasts from parallel control cultures (i).

Ensuing experiments involved mature dopaminergic neurons differentiated from neural progenitors generated from our iPSC lines. Specifically, neural progenitor cells were first produced from reprogrammed adult mouse tail-tip cells using a neuronal progenitor medium during Days 29–35 (**Figure 5A**-a). Then, from Day 36 to 50, these cells were further directed to differentiate into midbrain mature dopaminergic neurons using a dopamine neuronal differentiation medium (see Materials and Methods). At this point, the cells showed clear signs of forming mature neural networks (**Figure 5A**-b,c). Importantly, long-term observation of these cell lines until 6 months after the initial fibroblast cell reprogramming revealed that the cultured dopaminergic neurons still had a pronounced ability to form mature neuronal networks (**Figure 5A**-d). In contrast, at the 6-month mark, tail-tip fibroblast cells grown in control cultures (i.e., without cell reprogramming and differentiating media) maintained their original shape and failed to form any visible cellular networks (**Figure 5A**-e).

These significant developmental changes were characterized by greatly diminished expression of the early neuronal marker, DCX (**Figure 5A**-f), along with enhanced expression of the mature neuronal marker, MAP2 (**Figure 5A**-g). As well, these *in vitro* generated neurons expressed TH, the rate-limiting enzyme in dopamine synthesis (**Figure 5A**-h). Further evidence for a dopaminergic phenotype of the re-programmed neurons is evident from the ability of these cells to actually produce dopamine (**Figure 5A**-i). Indeed, concentrations of dopamine were increased in the re-programmed tail cells (364 pg/mg protein) compared to the original non-re-programmed tail tissue (169 pg/mg protein). The dopamine levels of the re-programmed cells approached those evident in actual dissected adult midbrain tissue (486 pg/mg protein). Together, these data are strongly indicative of a dopaminergic cell phenotype.

As a summary, **Figure 5B** presents the time course for the reprogramming of fibroblasts through progressive stages including clustered iPSCs (**Figure 5B**-a), followed by cellular outgrowths (**Figure 5B**-b), differentiation into neural progenitor cells (**Figure 5B**-c), dopaminergic neural progenitors (**Figure 5B**-d) and finally mature dopaminergic neurons (**Figure 5B**-e,f,g,h). The cellular morphology and series of network connections were easily visible and distinguishable from the original un-reprogrammed adult fibroblasts from parallel control cultures (**Figure 5B**-i).

### iPSC-DERIVED MATURE DOPAMINERGIC NEURONS SURVIVE FOLLOWING TRANSPLANTATION

**FIGURE 6:**
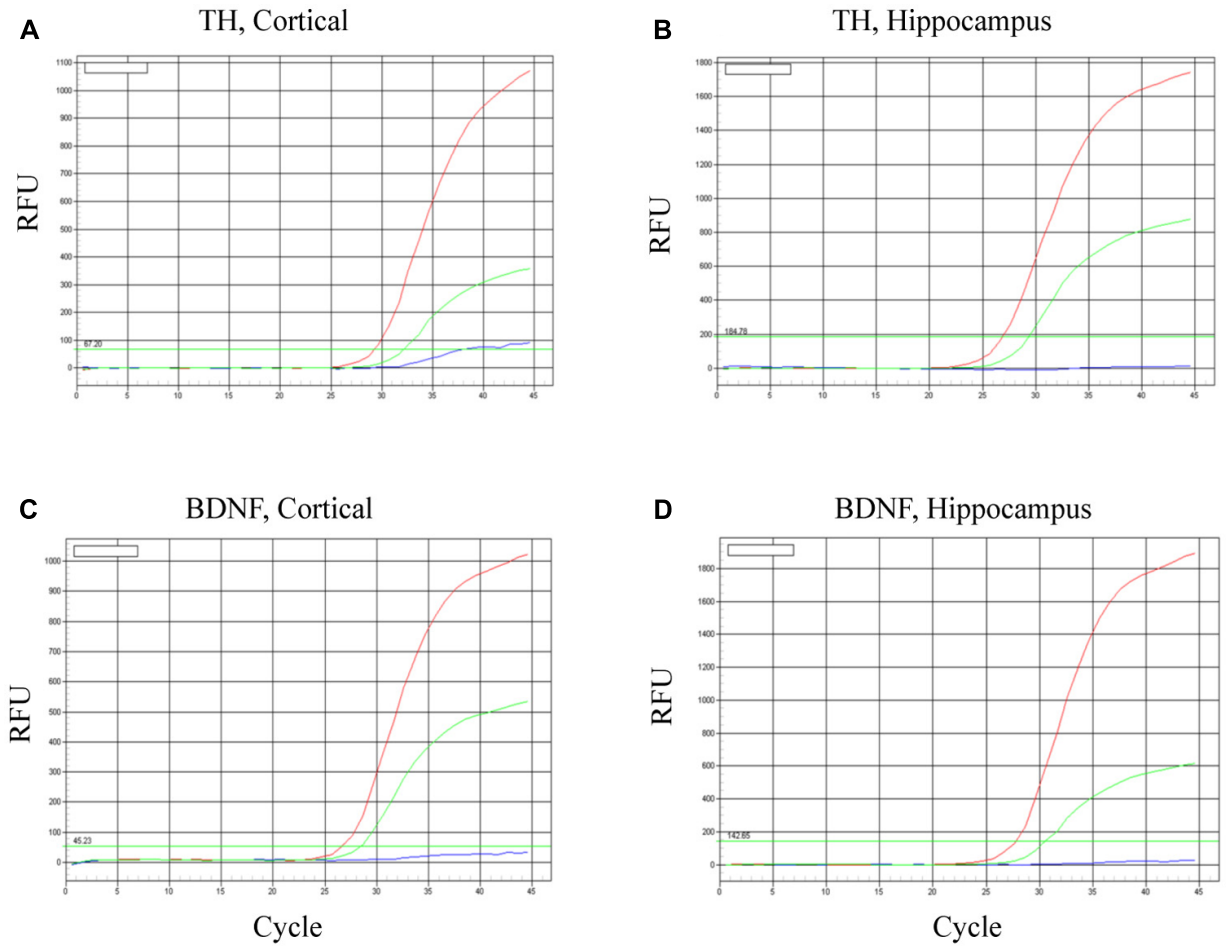
**Survival of iPSC-derived mature dopaminergic neurons *in vivo*.** Transplanted (i.e., intracerebroventricular infusion) neurons of an apparent dopaminergic phenotype (re-programmed tail cells after 50 days in differentiation medium) displayed clear elevations of TH and BDNF expression after 24 h. Panels **(A,B)** display TH mRNA, while **(C,D)** display BDNF mRNA within cortical and hippocampal dissected tissue. Within each of the four panels, identical patterns of gene expression were observed. Namely, the dopaminergic neurons (previously reprogrammed by vector 20866) plus transfected cDNA BDNF (red lines) induced the highest level of TH and BDNF expression, whereas dopaminergic neurons (previously reprogrammed by vector 20866) in the absence of BDNF cDNA had intermediate levels of expression (green lines) and the HBSS controls clearly had the as the lowest (background) gene expression (blue lines).

To evaluate neuronal survival and viability, TH and BDNF mRNA were determined in the cortex (**Figures 6A,C**) and hippocampus (**Figures 6B,D**). As predicted, in both brain regions BDNF mRNA was increased most robustly in mice transplanted with the BDNF-transfected neurons (red lines). Relative to controls (HBSS vehicle alone; blues lines), mice infused with mature dopaminergic neurons only (i.e., in the absence of BDNF; green lines) displayed intermediate elevations of TH mRNA and BDNF mRNA. These data suggest that the fibroblast-derived mature dopaminergic neurons can survive for at least for 24-h in mouse brain *in vivo*, during which time they appear to be capable of up-regulating TH and BDNF expression.

**FIGURE 7:**
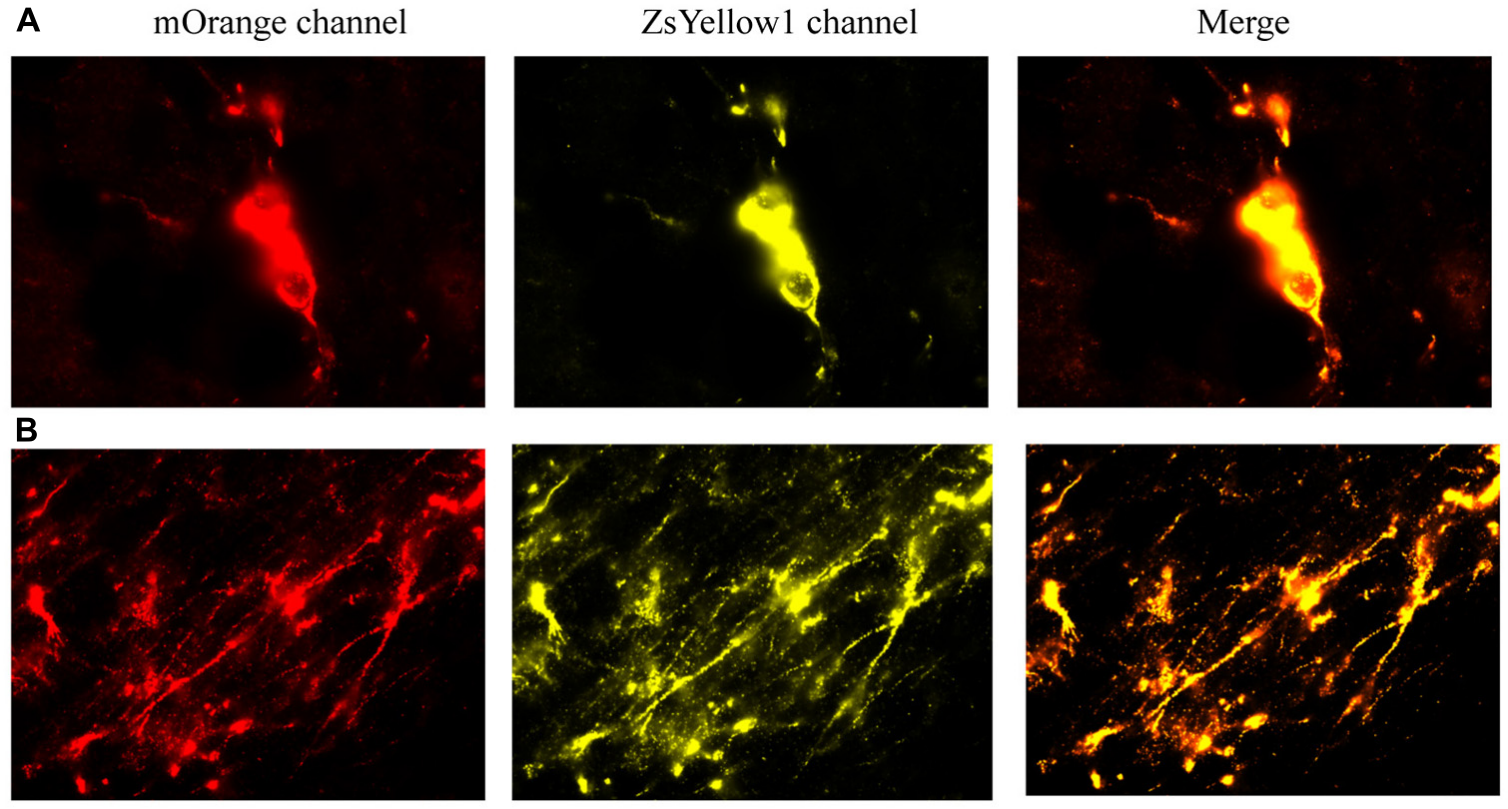
**The reprogrammed cells were directly imaged to observe the mOrange, which was linked to the 20866 vector, and ZsYellow1, which was linked to the cDNA BDNF.** Both fluorescent channels showed positive signals for the reprogramming vector (mOrange) and BDNF (ZsYellow) and these were highly co-localized (merge). Mice that received the non-reprogrammed control cells failed to display and positive cellular fluorescent staining and are hence, not shown. However, the upper **(A)** and lower **(B)** photomicrographs depict images of positive soma and fiber projections, respectively, from the vector 20866 + BDNF cDNA infused animals. Importantly, the specific genes were within the same reading frame and images were collected immediately adjacent to the site of infusion.

We first provide evidence that infused cells that had cDNA BDNF were actually still viable at 2 weeks following their central infusion (**Figure 7**). Specifically, slides were imaged for the vector 20866 biomarker using mOrange, and ZsYellow1 for BDNF. Clearly, a positive signal was observed for both fluorescent channels and the signals were highly co-localized, suggesting that the infused cells (at least a subset) still survived after 2 weeks *in vivo*. As shown in **Figure 7**, cellular bodies (upper panels) and fibers (lower panels) were apparent from the infused cells.

## DISCUSSION

The recently developed iPSC-generating technologies allow for the transplantation of stem cells (or even mature neurons) derived from one’s own somatic cells. This could conceivably pave the way for the advent of patient- and disease-specific stem cell treatments (i.e., for personalized medicine). Although most current iPSC-generating systems are virus-based, the feasibility of generating iPSCs from fibroblasts using a single excisable non-viral vector containing a 4-gene reprogramming cassette is beginning to be assessed. In the present investigation we have taken the “next step” by demonstrating that iPSC-derived cells transformed into neural cells had functional consequences in the context of stressor exposure, and that some of these effects were further modified when the iPSC-derived cells were engineered to express BDNF.

In the present investigation we were particularly interested in using iPSC-derived cells as a means of stably delivering BDNF into the brain and assessing whether this would elicit “anti-stress” effects that could be beneficial for modulating stressor-induced pathology. Indeed, intracerebroventricular infusion of iPSC-derived neural progenitors (i.e., DCX expressing immature neurons) did blunt the impact of LPS + chronic stressor exposure upon neurogenesis and corticosterone levels, two key mediators of stressor related pathology (Tanti and Belzung, 2013). Moreover, in addition to the neural progenitor cells, we were able to create iPSC-derived mature dopamine producing neurons that survived, at least transiently, following *in vivo* infusion. However, it remains to be determined if these more mature neurons can usefully integrate into endogenous neural circuits and impart long-term functional changes.

Stressor exposure has been strongly linked to the development of psychiatric conditions (Golden et al., 2011), and immune challenges were shown to further augment the impact of stressors (Gibb et al., 2011; Hayley, 2011). Moreover, in the case of major depression, structural and functional changes have been documented in discrete brain regions, including the hippocampus and amygdala (van Eijndhoven et al., 2009; Sheline et al., 2012; Bielau et al., 2013; Vassilopoulou et al., 2013). Yet, there are no known clinically available treatments that specific target such potential neuronal or synaptic pathology, rather than simply managing neurotransmitter disturbances. Intriguingly however, one preclinical study reported that intracerebroventricularly infused mesenchymal stem cells migrated to the hippocampus and promoted antidepressant-like behavioral effects, along with increased neurogenesis in adult rodents (Tfilin et al., 2010).

Curiously, in the present investigation central infusion of iPSC-derived neural cells did not affect hippocampal neurogenesis, although it did reverse the LPS + stressor-induced reduction of SVZ neurogenesis. It is likely that the SVZ region was selectively affected given its proximity to the site of infusion. Most importantly, BDNF appeared to be a key factor in promoting SVZ neurogenesis, as infusion of the iPSC derived cells in the absence of enhanced BDNF expression had no significant effect. As well, the fact that infusion of iPSC derived cells modestly blunted (∼30%) the corticosterone elevation induced by LPS + stressor exposure is tantalizing and raises the possibility that the infused cells may have been influencing hippocampal or conceivably even hypothalamic neurons, thereby modulating their respective inhibitory or excitatory control of CRH neuronal activity. Of course, another possibility that warrants consideration is that the infused iPSC derived cells induced non-specific compensatory responses that serve to keep stressor reactivity in check, or that the exogenous cells might have influenced processes aligned with habituation to the ongoing stressor.

Taken together, the current findings represent a starting point for future studies aimed at the possibility of combining stem cells (as a cell replacement therapy and gene delivery tool) and BDNF (as a trophic factor) as a potential strategy for dealing with stressor or immune challenge induced neuronal pathology. In particular, long-term stable BDNF expression in specially tailored neuron cells could potentially stimulate neurogenesis and repair, contribute to neuroprotection and possibly modulate the impact of psychological stressors (Greisen et al., 2005; Nomura et al., 2005; Anisman et al., 2008; Hayley, 2011). We urge caution in interpreting these data as we are uncertain as to whether the exogenously administered iPSC derived cells: (1) actually integrated into the existing neural circuitry or (2) induced endogenous factor(s), which in turn affected the outcomes, including HPA and neurogenic responses. Indeed, further studies are required to verify whether the infused cells were the sole source of increased central BDNF and if iPSC-derived cells could permanently influence neural circuitry. That said, the present technical approach is novel and deserves further study concerning its potential applicability for a variety of animal models of disease. Ultimately, it is hoped that future work can identify the conditions that favor beneficial additive or even synergistic outcomes with the over-expression of trophic factors in iPSC-derived neural cells.

## AUTHOR CONTRIBUTIONS

Conception and design of the work: Gele Liu, Shawn Hayley, and Hymie Anisman; Experiments and acquisition of data for the work: Gele Liu, Nazneen Rustom, Jessica Bobyn, Chris Rudyk, and Meagan Osborn; Analysis and interpretation of data for the work: Gele Liu, and Shawn Hayley; Drafting the work: Gele Liu; Revising it critically for important intellectual content: Shawn Hayley and Darcy Litteljohn; Final approval of the version to be published: Shawn Hayley, and Hymie Anisman.

## Conflict of Interest Statement

The authors declare that the research was conducted in the absence of any commercial or financial relationships that could be construed as a potential conflict of interest.
